# Mélanome endobronchique

**Published:** 2010-08-09

**Authors:** Serraj Mounia, Znati Kaoutar, Nfissi Loubna, Meziane Mériem, Choumi Ilham, Amara Bouchra, El Biaze Mohammed, Amarti Afaf, Zohra Mernissi Fatima, Chakib Benjelloun Mohammed

**Affiliations:** 1 Service de pneumologie, CHU Hassan II, Fès, Maroc; 2 Service d’anatomopathologie, CHU Hassan II, Fès, Maroc; 3 Service de dermatologie, CHU Hassan II, Fès, Maroc

**Keywords:** Mélanome endobronchique, mélanome pulmonaire, métastase endobronchique, bronchoscopie

## Abstract

Le mélanome malin a un potentiel métastatique important. Les métastases pulmonaires du mélanome sont communes cependant la localisation endobronchique reste rare et pose le problème de son origine primitive ou secondaire.

Nous rapportons le cas d’un mélanome pulmonaire qui présente des particularités intéressantes: une lésion cutanée présumée primitive totalement régressive, la présentation radio clinique mimant parfaitement un cancer bronchique primitif, un aspect endoscopique bourgeonnant et grisâtre dont l’étude histologique a permis de poser le diagnostic, une agressivité tumorale avec une extension intracardiaque et bourgeon tumoral intra cavitaire.

A travers cette observation, les auteurs étudient les caractéristiques radio-cliniques pouvant distinguer le mélanome pulmonaire primitif du secondaire; la localisation endobronchique avec une revue de la littérature sur les métastases endo bronchiques; le bilan d’extension à entamer en cas de mélanome pulmonaire ainsi que les difficultés thérapeutiques posés par ce type de lésion dont le pronostic reste péjoratif.

## Introduction

La découverte d’un mélanome pulmonaire est une situation rarement rencontrées. Il faut alors affirmer la nature secondaire du mélanome pulmonaire, situation la plus fréquente, ou plus rarement le caractère primitif fait exceptionnel vue l’absence de mélanocytes au niveau du tractus respiratoire [1,2].

La métastase endobronchique est beaucoup plus rare, les métastases pulmonaires étant généralement parenchymateuses. La première description de métastase endobronchique remonte à 1890 [3,4]. Dans les séries autopsiques 2% des tumeurs solides présentent des métastases endobronchiques [5,6]. Le mélanome est une tumeur fréquente avec un fort potentiel métastatique. Les poumons sont souvent siège de métastases de tumeur extra thoracique, celles-ci sont généralement parenchymateuses.

Nous rapportons un cas clinique qui illustre parfaitement le caractère agressif et métastatique d’un mélanome cutané régressif. La présentation radio-clinique de cette métastase pulmonaire mimait parfaitement le carcinome bronchique primitif en se présentant comme une masse centrale avec une expression endobronchique en bourgeon et une atteinte intracardiaque. Nous étudierons également les difficultés diagnostiques et thérapeutiques de ce cas particulier.

## Patient et observation clinique

L’accord du patient a été obtenu pour reporter ce cas. Monsieur ML, âgé de 65 ans, agriculteur de profession, hospitalisé au service des maladies respiratoires pour des épisodes répétés d’hémoptysies de faible abondance apparues 15 jours avant et accompagnés de douleurs thoraciques droites. L’examen clinique trouvait un patient en bon état général avec un index OMS 1, un syndrome de condensation basale droit ainsi qu’une macule hyper pigmentée mesurant 2,5 cm sur 2 cm, bien limitée à bords réguliers siégeant au niveau de la partie supérieure de la joue droite. Cette lésion évoquait en premier lieu un mélanome.

**Figure 1:**
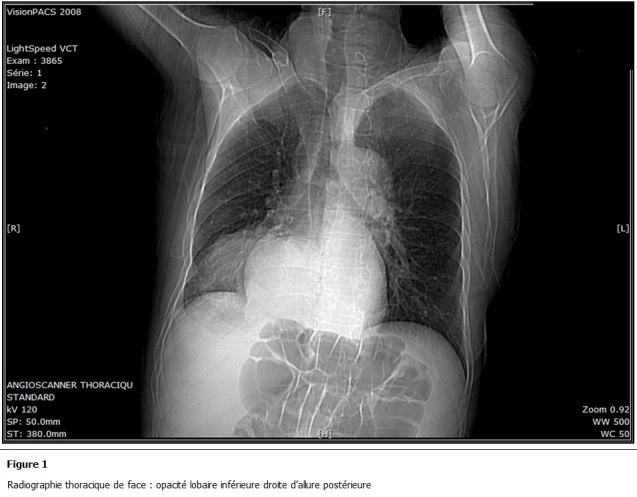
Radiographie thoracique de face: opacité lobaire inférieure droite d’allure postérieure

La radiographie thoracique de face (figure 1) trouvait une opacité intéressant le tiers inférieur de l’hémithorax droit dense et homogène n’effaçant ni le bord droit du cœur ni la coupole diaphragmatique.

**Figure 2:**
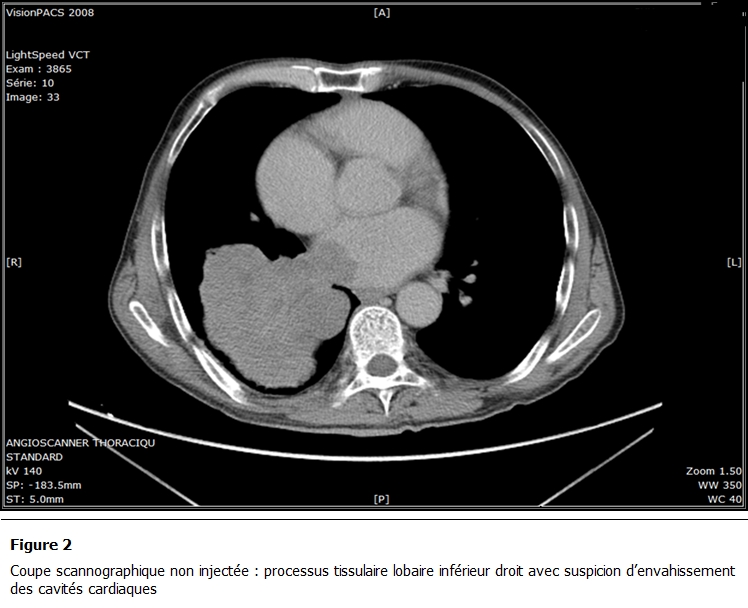
Coupe scannographique non injectée: processus tissulaire lobaire inférieur droit avec suspicion d’envahissement des cavités cardiaques

La TDM Thoracique avec injection de produit de contraste (figure 2 et 3) objectivait une volumineuse lésion tissulaire hétérogène polylobée au niveau de la pyramide basale droite. Cette lésion s’étendait au cœur. Les coupes réalisées après injection de produit de contraste mettaient en évidence l’extension tumorale à l’oreillette gauche. Celle-ci était siège d’un volumineux bourgeon endoluminal.

La fibroscopie bronchique (figure 4) retrouvait un bourgeon grisâtre au niveau de la segmentaire postéro-basale de la pyramide basale droite.

Des biopsies du bourgeon étaient réalisées, ainsi qu’une biopsie de la lésion cutanée.

L’étude histologique des biopsies bronchiques a conclu à une localisation bronchique d’un mélanome; l’étude anatomopathologique de la biopsie cutanée retrouvait également un mélanome sur mélanose de Dubreuil en régression faisant 0,5 mm d’épaisseur selon Breslow (figures 5 et 6). Le bilan d’extension retrouvait également une métastase cérébrale (figure 7).

L’attitude thérapeutique s’est basée sur une radiothérapie cérébrale associée à une chimiothérapie.

**Figure 3:**
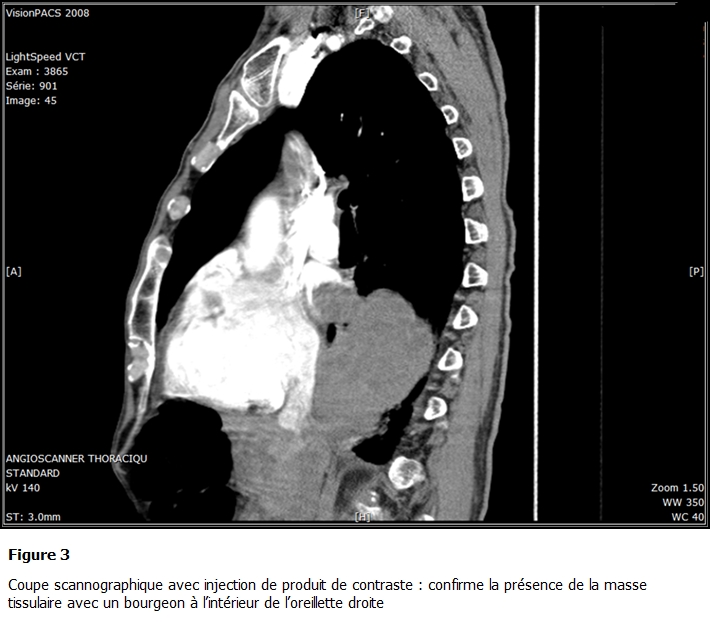
Coupe scannographique avec injection de produit de contraste: confirme la présence de la masse tissulaire avec un bourgeon à l’intérieur de l’oreillette droite

**Figure 4:**
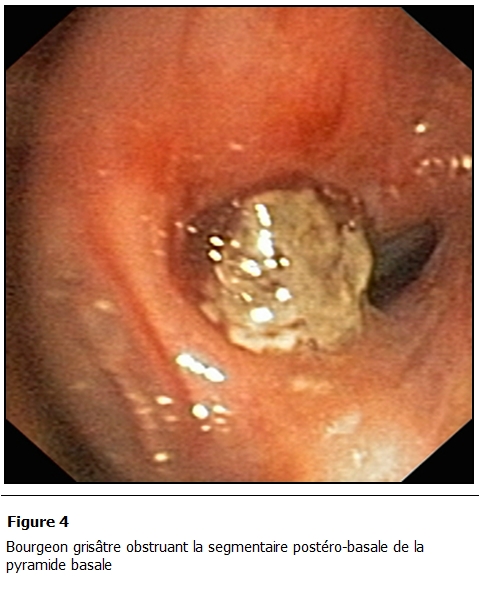
Bourgeon grisâtre obstruant la segmentaire postéro-basale de la pyramide basale

**Figure 5:**
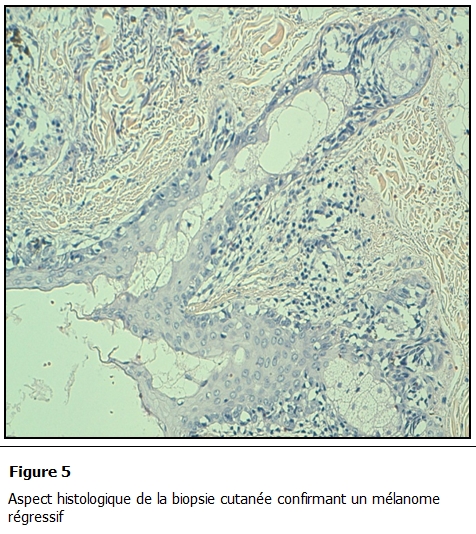
Aspect histologique de la biopsie cutanée confirmant un mélanome régressif

**Figure 6:**
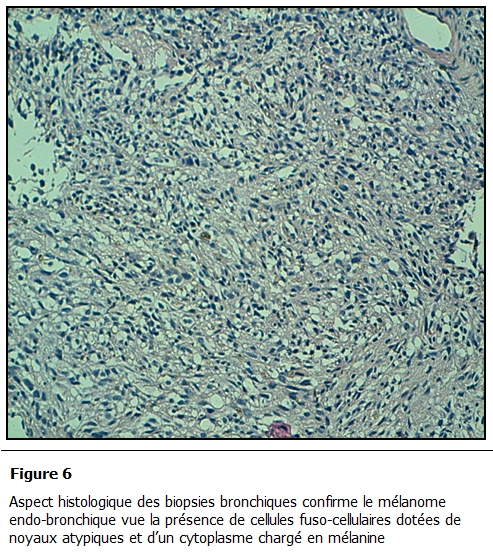
Aspect histologique des biopsies bronchiques confirme le mélanome endo-bronchique vue la présence de cellules fuso-cellulaires dotées de noyaux atypiques et d’un cytoplasme chargé en mélanine

**Figure 7:**
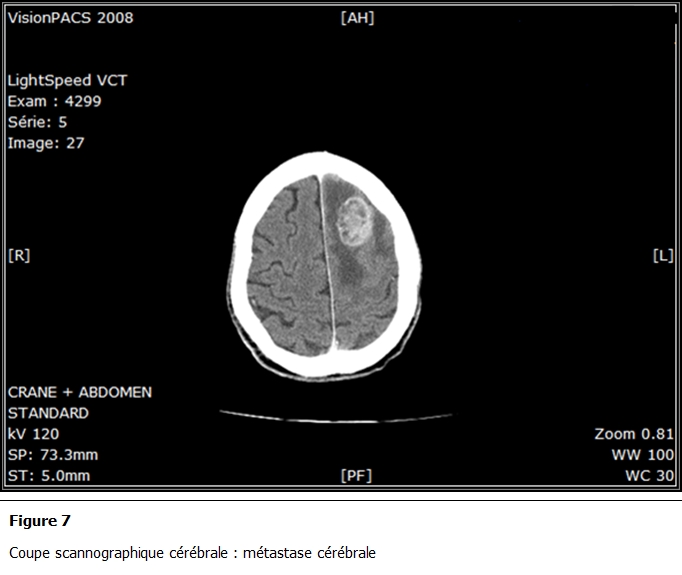
Coupe scannographique cérébrale: métastase cérébrale

## Discussion

Le poumon est un site métastatique classique de tumeurs extra-thoraciques ; cependant les métastases endobronchiques sont des localisations secondaires rares. Braman et Whitcomb [5,7] ont limité les métastases endobronchiques aux lésions intéressant les bronches souches et lobaires, ils ont rapportés une incidence de 2% dans des séries autopsiques.

Les cancers primitifs sont majoritairement de siège mammaire, puis colorectal, rénal, vésical. L’origine cutanée de la tumeur primitive est plus rarement rapportée. La distinction entre mélanome pulmonaire primitif et localisation secondaire d’un mélanome est souvent difficile quand la lésion pulmonaire est unique et se présente comme une tumeur endobronchique. Des mélanomes métastatiques de primitifs inconnus ainsi que des métastases de mélanomes complètement régressifs, comme le cas de notre patient, ont été rapportés [8,9]. Dans notre cas ce n’est pas primitif et non plus totalement régressif. La distinction entre ces métastases à développement endobronchique de mélanome ayant totalement régressé et mélanome pulmonaire primitif s’avère alors impossible et fait toujours l’objet de discussion et de contentieux.

Les mécanismes évoqués dans le développement de métastases endobronchiques sont : localisation métastatique directement endobronchique, métastase parenchymateuse pulmonaire avec extension endobronchique secondaire, extension endobronchique d’une masse médiastinale, dissémination lymphatique [10].

Les symptômes clinico-radiologiques des métastases endobronchiques peuvent prêter à confusion avec un carcinome bronchique primitif. Les symptômes d’appel sont la toux et l’hémoptysie rapportées dans 41 – 62% [11]. Le diagnostic repose sur les biopsies per endoscopiques, il est actuellement facilité par les techniques d’immunomarquage sur biopsies bronchiques.

## Conclusion

Le pronostic des métastases endobronchiques en général et particulièrement du mélanome métastatique est sombre. Le seul traitement reconnu est l’exérèse chirurgicale, il suppose l’absence d’autres localisations secondaires et la possibilité technique chirurgicale. La chimiothérapie et la radiothérapie n’ont pas fait leur preuve. Actuellement plusieurs techniques endoscopiques sont utilisées dans un but palliatif afin de lever l’obstruction bronchique (électrocoagulation, laser Yag, etc.). Ces procédures semblent également intéressantes dans la prévention des hémoptysies, des atélectasies et des pneumonies.

## Conflit d’intérêts

Les auteurs ne déclarent aucun conflit d’intérêts.

## Contribution des auteurs

Le patient a été initialement hospitalisé au service de pneumologie pour bilan d’une opacité pulmonaire; il a été pris en charge par l’équipe de pneumologie (Dr Mounia Serraj, Loubna Nfissi, Ilham Choumi, Bouchra Amara), il a alors bénéficié des explorations endoscopiques pneumologiques et du bilan d’extension, bilan pré-thérapeutique et prise en charge thérapeutique. La lecture des examens anatomopathologiques a été effectuée au sein du service d’anatomie-pathologie par Dr Kaoutar Znati et Dr Amarti, et il a également bénéficié d’avis dermatologique et de biopsies cutanées réalisées par Dr Méziane er Dr Mernissi.
